# Functional Neuroplasticity in the Nucleus Tractus Solitarius and Increased Risk of Sudden Death in Mice with Acquired Temporal Lobe Epilepsy

**DOI:** 10.1523/ENEURO.0319-17.2017

**Published:** 2017-10-30

**Authors:** Isabel D. Derera, Brian P. Delisle, Bret N. Smith

**Affiliations:** 1 Department of Physiology, College of Medicine, University of Kentucky, Lexington, KY 40536; 2 Epilepsy Center, University of Kentucky, Lexington, KY 40536

**Keywords:** Autonomic, brainstem, epilepsy, EPSC, GABA, vagus

## Abstract

Sudden unexpected death in epilepsy (SUDEP) is the leading cause of death in individuals with refractory acquired epilepsy. Cardiorespiratory failure is the most likely cause in most cases, and central autonomic dysfunction has been implicated as a contributing factor to SUDEP. Neurons of the nucleus tractus solitarius (NTS) in the brainstem vagal complex receive and integrate vagally mediated information regarding cardiorespiratory and other autonomic functions, and GABAergic inhibitory NTS neurons play an essential role in modulating autonomic output. We assessed the activity of GABAergic NTS neurons as a function of epilepsy development in the pilocarpine-induced status epilepticus (SE) model of temporal lobe epilepsy (TLE). Compared with age-matched controls, mice that survived SE had significantly lower survival rates by 150 d post-SE. GABAergic NTS neurons from mice that survived SE displayed a glutamate-dependent increase in spontaneous action potential firing rate by 12 wks post-SE. Increased spontaneous EPSC frequency was also detected, but vagal afferent synaptic release properties were unaltered, suggesting that an increase in glutamate release from central neurons developed in the NTS after SE. Our results indicate that long-term changes in glutamate release and activity of GABAergic neurons emerge in the NTS in association with epileptogenesis. These changes might contribute to increased risk of cardiorespiratory dysfunction and sudden death in this model of TLE.

## Significance Statement

Sudden unexpected death in epilepsy (SUDEP) accounts for ∼17% of deaths in individuals with epilepsy, but the reasons underlying this increased risk are not known. Most research on SUDEP has focused on genetic models of epilepsy, identifying seizure-related changes in autonomic function as a contributing factor to sudden death in these models, but little is known about SUDEP in models of acquired epilepsy. Here, we show that mice die suddenly and unexpectedly, and excitability in brainstem neurons that regulate cardiorespiratory function is chronically increased, in a mouse model of acquired epilepsy. These results are the first to identify a model of SUDEP in acquired epilepsy and to demonstrate functional changes in brainstem circuitry in response to epileptogenesis.

## Introduction

Sudden unexpected/unexplained death in epilepsy (SUDEP) occurs when an individual with epilepsy who is otherwise healthy dies suddenly for unknown reasons (Annegers, 1997; Nashef, 1997; Nashef et al., 2012; Tolstykh and Cavazos, 2013). For epilepsy patients, the risk of sudden, unexpected death is >20-fold higher than in the general population and accounts for ∼17% of epilepsy-related deaths (Nashef, 1997; Kalume et al., 2013; Tolstykh and Cavazos, 2013), so it is imperative to elucidate its underlying mechanisms. Patients with longstanding epilepsy characterized by frequent generalized tonic-clonic seizures that are relatively poorly controlled are at highest risk (Tolstykh and Cavazos, 2013; Thurman et al., 2014). Patients with temporal lobe epilepsy (TLE) represent ∼60% of all epilepsies, and seizures are medically intractable in ∼30% of these patients, making this the largest population at risk of SUDEP, yet mechanisms underlying increased SUDEP risk have not been identified in animal models of acquired TLE. Peri-ictal, centrally originating or peripheral autonomic irregularities leading to cardiorespiratory collapse may be the immediate cause of death in SUDEP (Ryvlin et al., 2013), but few studies have been aimed at identifying mechanisms underlying this autonomic failure. Seizures can increase activity of neurons in brainstem autonomic areas, independently of physical activity or peripheral metabolic influences (Kanter et al., 1995; Takakura et al., 2011), and autonomic irregularities often develop over time in individuals with epilepsy, implicating central or peripheral autonomic reactive neuroplasticity as a potential driver of increased SUDEP risk in patients and in rodent epilepsy models (Glasscock et al., 2010; Massey et al., 2014; Biet et al., 2015). Thus, recurrent seizures might induce changes in central or systemic physiologic functions that increase the risk for sudden death.

The brainstem vagal complex is the principal neural center mediating parasympathetic visceral regulation. Within the vagal complex, neurons of the nucleus tractus solitarius (NTS) receive viscerosensory information via vagal afferents and project their axons to preganglionic parasympathetic motor neurons in the dorsal motor nucleus of the vagus (DMV) and the nucleus ambiguus (NA; Andresen and Kunze, 1994; Doyle and Andresen, 2001; Wang et al., 2001a, b; Davis et al., 2004; Travagli et al., 2006; Glatzer et al., 2007; Bailey et al., 2008), as well as to brainstem and hypothalamic areas responsible for premotor sympathetic regulation and respiratory reflexes (Takenaka et al., 1995; Fontes et al., 2001; Irnaten et al., 2001; Bonham et al., 2006; Affleck et al., 2012; Zoccal et al., 2014). Inhibitory GABAergic NTS neurons participate in vagal reflexes and prominently regulate parasympathetic output (Davis et al., 2004; Travagli et al., 2006). Evidence from genetic epilepsy models suggests that epilepsy-related alterations in peripheral or central vagal function contribute to cardiorespiratory collapse and SUDEP (Glasscock et al., 2010, 2012; Cheah et al., 2012; Kalume et al., 2013; Aiba and Noebels, 2015). Central vagal circuit plasticity is prominent in disease states that affect autonomic homeostasis (Mei et al., 2003; Bach et al., 2015; Bhagat et al., 2015; Boychuk et al., 2015), and seizure-related derangement of central vagal system function has been briefly described, manifesting as increased likelihood of spreading depolarization in the NTS (Aiba and Noebels, 2015). Reactive neuroplasticity in the central vagal complex, however, has not been investigated in animals with acquired TLE.

We used the pilocarpine-induced status epilepticus (pilo-SE) model of TLE in mice (Shibley and Smith, 2002; Borges et al., 2003; Winokur et al., 2004; Gröticke et al., 2007; Bhaskaran and Smith, 2010a, b; Hunt et al., 2013) to identify long-term changes in NTS neuron function coinciding with the development of TLE. Because the NTS is the primary integration center for cardiorespiratory reflexes, and GABA neurons in particular are principal participants in vagal reflex activity (Glatzer et al., 2007; Bailey et al., 2008), increased excitability of these neurons would be consistent with an increased propensity for central autonomic failure that could lead to SUDEP in TLE. We tested the hypothesis that reactive plasticity of GABAergic circuitry in the NTS emerges over time in mice that survive pilocarpine-induced SE.

## Methods

### Animals

Four- to six-week-old age-matched male Hsd:ICR (CD-1; Envigro-Harlan) and GIN (green inhibitory neuron) mice (Oliva et al., 2000), which express EGFP under the control of the mouse GAD67 promoter [FVB-Tg(GADGFP)4570Swn/J; The Jackson Laboratory] were used. All mice were housed under a 14-h light/10-h dark cycle in an Association for Assessment and Accreditation of Laboratory Animal Care International (AALAC)-approved facility. Water and food were available *ad libitum*. The University of Kentucky Institutional Animal Care and Use Committee approved all procedures.

### Pilocarpine treatment and seizure assessment

Mice were administered an intraperitoneal (i.p.) injection of the muscarinic receptor antagonist, methylscopolamine (1 mg/kg in 0.9% NaCl) 20 min before an i.p. injection of pilocarpine (280–285 mg/kg in 1 ml 0.9% NaCl). The methylscopolamine served to block peripheral effects of pilocarpine. Age-matched control mice received a comparable amount of vehicle 20 min after the methylscopolamine. Behavioral observations of seizures lasted 2 h after pilocarpine, and seizures were evaluated according to a modified Racine scale (Racine, 1972), using categories 1–5, with 5 being the most severe. Categories 1 and 2 (i.e., facial automatisms, tail stiffening, and wet-dog shakes) were considered together to reduce subjectivity. All pilocarpine-treated mice displayed some or all of these behaviors. Category 3 (i.e., low-intensity tonic-clonic seizures marked by unilateral forelimb myoclonus), category 4 (i.e., the addition of bilateral forelimb myoclonus and rearing), and category 5 (i.e., bilateral fore- and hindlimb myoclonus and transient loss of posture) were considered to be general convulsive seizures. Category 3–5 seizures were typically 30–90 s in duration and were separated by periods of relative inactivity of variable duration. A mouse that experienced a minimum of 3 convulsive (category 3–5) seizure events within 2 h postinjection were considered to have undergone SE, as >90% these mice go on to develop spontaneous seizures (Shibley and Smith, 2002; Winokur et al., 2004; Bhaskaran and Smith, 2010a, b). In addition to standard diet, mice were given water-moistened food and a 5% glucose solution in a Petri dish inside the cage for 4 d after SE. A subset of mice was maintained for up to 150 days to assess for survival. Other mice were used for *in vitro* electrophysiological recordings at 1, 6, or 12 wks post-SE. A subset of mice was monitored for behavioral seizure activity for 6 h during the week (i.e., 2 h/d, three nonconsecutive days/wk) before their use in electrophysiological recordings. Spontaneous seizure activity was rated using the modified Racine scale by an observer that was blinded to the experimental groups. Only behavioral seizures at or above category 2 and lasting longer than 10 s were tabulated (Shibley and Smith, 2002; Hunt et al., 2009; Butler et al., 2015).

### Brainstem slice preparation

On-cell and whole-cell patch-clamp recordings were performed in GABAergic NTS neurons from brainstem slices in GIN mice. Mice were deeply anesthetized by isofluorane inhalation and then decapitated while anesthetized. The brain was removed and blocked on an ice-cold stand, and the brainstem was glued on a platform for sectioning in the coronal plane. Transverse (coronal) slices (300 μm) from the caudal brainstem were made in cold (0–2°C) oxygenated (95% O_2_/5% CO_2_) artificial cerebral spinal fluid (aCSF) using a vibrating microtome (Vibratome Series 1000; Technical Products International) and transferred to a holding chamber. These brainstem slices contain the NTS and preserve many intact intrinsic synaptic connections, including from primary vagal afferents in the solitary tract (ST) among vagal complex neurons (Doyle and Andresen, 2001; Glatzer et al., 2003; Davis et al., 2004; Bach and Smith, 2012). The aCSF contained (in mm): 124 NaCl, 3 KCl, 2 CaCl_2_, 1.3 MgCl, 1.4 NaH_2_PO_4_, 26 NaHCO_3_, and 11 glucose (pH 7.2–7.4). For recordings, a single brain slice was transferred to a chamber mounted on a fixed stage under an upright microscope (BX51WI; Olympus), where it was superperfused with continuously oxygenated and warmed (30°–32°C) aCSF.

### Electrophysiological recordings

On-cell and whole-cell patch-clamp recordings were obtained in GABAergic caudal NTS neurons identified by EGFP expression in FVB male mice. Recording pipettes were pulled from borosilicate glass (open tip resistance of 3–5 MΩ; King Precision Glass Co.). The pipette solution for recordings contained (in mm): 130 K^+^-gluconate, 1 NaCl, 5 EGTA, 10 HEPES, 1 MgCl_2_, 1 CaCl_2_, 3 KOH, and 2 ATP. GABAergic neurons in the NTS were targeted for recording under a 40× water-immersion objective with epifluorescence (Olympus). Electrophysiological signals were obtained using Multiclamp 700B amplifier (Molecular Devices), low-pass filtered at 2–3 kHz, digitized at 20 kHz, and recorded onto a computer (Digidata 1440A, Molecular Devices) using pClamp 10.2 software (Molecular Devices). Seal resistance was typically 2–5 GΩ; series resistance was <25 MΩ (mean ± SEM = 16.21 ± 5.00 MΩ, *n* = 218 cells) and was monitored periodically throughout recordings. Electrophysiology data were not further analyzed if the series resistance changed by >20% during the recording.

Spontaneous action potential firing in GABAergic NTS neurons was measured using on-cell patch-clamp recordings. Cells were recorded at resting membrane potential, and action potential frequency was calculated over a 2-min segment of continuous firing for each cell. Spontaneous, miniature, and evoked excitatory postsynaptic currents (sEPSCs, mEPSCs, and eEPSCs) were examined in whole-cell recordings at a holding potential of –70 mV. The Na^+^ channel blocker, tetrodotoxin (TTX, 1 μm, Alamone Labs) was added to the aCSF for ∼10 min before recordings of mEPSCs. Resting membrane potential was measured in current-clamp mode by measuring the mean resting membrane potential averaged over a 30-s period with no spontaneous action potential firing. If there was spontaneous action potential firing, resting membrane potential was calculated by averaging portions between action potentials. Input resistance was calculated by plotting the linear portion of the current−voltage curve and calculating the slope. A platinum-iridium concentric bipolar electrode (125 μm diameter; FHC) was placed on the ST to activate vagally evoked EPSCs (Glatzer and Smith, 2005; Glatzer et al., 2007). Sets of five current pulses (30–50 μA; 400 μs) were delivered at 50 Hz. The stimulus intensity was adjusted so that eESPCs occurred >80% of the time in GABAergic NTS neurons when stimulation was applied to the ST. Stimulus sweeps were included only if an eEPSC was elicited after each stimulation in that sweep.

### ECG telemetry

*In vivo* telemetry was used to evaluate chronic changes in heart rate and two measures of heart rate variability (HRV), the standard deviation between the N-to-N interval (SDNN) and root mean squared of the standard deviation (RMSSD), before and after methylscopolomine and pilocarpine or vehicle injection, as occurs in rats (Metcalf et al., 2009b; Bealer et al., 2010; Biet et al., 2015). Mice were anesthetized with 2.0% isofluorane in 100% O_2_ at 0.5 l/min, and telemetry units (model ETA-F10; Data Sciences International) were implanted subcutaneously. The transmitter body was placed on the right flank with the positive lead near the right pectoral muscle and the negative lead on the left abdomen. The leads were secured by being embedded into the fascia under the skin. Mice were housed individually and allowed to recover for 14 d postsurgery before pilocarpine or vehicle treatment. During recovery, mice were housed in an AALAC-approved satellite facility in which all ECG recordings were conducted, where they remained for the duration of the 12-wk recording period. To minimize disturbance and stress to the animals, implanted telemeters were switched on 24 h before each data collection period. Data were recorded for 24 h pre-pilocarpine or vehicle injection, 24 h after injection, and for 24-h periods at 6 and 12 wks after injection. ECG data were collected and analyzed with DSI DataQuest A.R.T. 4.31 and Ponemah 6.10 telemetry software. Data were acquired at a sampling rate of 1000 Hz, which is the standard rate used for mice and results in a smooth physiologic signal when the ECG waveforms are graphed; the telemetry device used a factory preset sampling rate of 200 Hz. No low-pass or high-pass filtering was applied during data acquisition. For ECG waveform analysis, the software was set to use a 40% QRS detection threshold (percentage of the largest derivative peak in a QRS segment resulting in an R that satisfies the minimum heart rate criteria), a minimum R deflection of 0.25 mV, a maximum heart rate of 1000 bpm, and a minimum heart rate of 80 bpm.

Average heart rate and HRV were calculated from data from 1-h recording periods, as described previously (Metcalf et al., 2009b; Ho et al., 2011; Schroder et al., 2015); all recordings were performed during seizure-free periods. The RR interval was manually examined and filtered for abnormal beats by sorting the RR intervals from shortest to longest and deleting cycles that were two standard deviations from the average RR interval, and the ECG channel was subsequently reanalyzed by setting upper and lower limits on RR values (Thireau et al., 2008). Areas of the recording that contained skipped beats or loss of signal were also deleted. The remaining cycles were then averaged to comprise the NN interval, which was then used to calculate the SDNN and RMSSD. The SDNN was calculated by taking the square root of the averaged NN interval. The RMSSD was calculated with the following steps: (1) the difference between the NN interval and delayed NN interval was squared; (2) the squared difference was summed; (3) the number of NN intervals was counted; and (4) the sum of the difference squared was divided by the count of NN intervals. RMSSD is reported as the square root of this value.

### Data analysis

A Kaplan–Meier survival curve was generated to assess survival rates in pilo-SE and control mice up to 150 d postinjection, using a log-rank (Mantel–Cox) test to assess statistical significance. On-cell recordings of spontaneous action potentials (2-min continuous recording) were examined with Clampfit 10.2 (Molecular Devices) to measure the frequency of spontaneous action potentials. Spontaneous and miniature EPSCs were analyzed with MiniAnalysis (Synaptosoft). An unpaired *t* test was used to compare mean action potential firing, sEPSC or mEPSC frequency and amplitude, resting membrane potential, input resistance, paired-pulse ratio (PPr), and frequency-dependent depression between age-matched vehicle or pilocarpine treated mice 1, 6, and 12 wks posttreatment. Two-way ANOVA (Tukey’s *post hoc*) was used to compare heart rate and heart rate variability in control and pilo-SE mice over a 12-wk electrocardiography recording period. Statistical measures were performed with Prism (GraphPad). Data are presented as mean ± SEM, and statistical significance was set at *p* < 0.05 for all measurements. Table 1 indicates the tests used for each assessment and includes confidence intervals for each statistical measurement.

**Table 1.** Statistical table

Outcome measure	Data structure	Type of test	Confidence interval
a. Survival curve	Nominal data, nonnormal distribution	Log-rank (Mantel–Cox)	0.03526 to 0.3404
b. Action potential firing frequency ACSF	Week 1: normal distribution Week 6: normal distribution Week 12: normal distribution	Unpaired *t* test	Week 1: 0.83 to 3.23 Week 6: 0.02 to 2.42 Week 12: 0.01 to 4.10
c. Action potential firing frequency KYN	Week 1: normal distribution Week 6: normal distribution Week 12: normal distribution	Unpaired *t* test	Week 1: –2.28 to 4.61 Week 6: –1.52 to 1.24 Week 12: –2.46 to 1.90
d. sEPSC frequency	Week 1: normal distribution Week 6: normal distribution Week 12: normal distribution	Unpaired *t* test	Week 1: 1.01 to 5.74 Week 6: 0.03 to 2.41 Week 12: 0.12 to 2.30
e. sEPSC amplitude	Week 1:normal distribution Week 6:normal distribution Week 12:normal distribution	Unpaired *t* test	Week 1: –4.88 to 6.12 Week 6: –3.93 to 3.56 Week 12: –7.66 to 1.72
f. mEPSC frequency	Week 1: normal distribution Week 6: normal distribution Week 12: normal distribution	Unpaired *t* test	Week 1: –0.54 to 1.52 Week 6: 0.65 to 2.74 Week 12: 0.30 to 1.81
g. mEPSC amplitude	Week 1: normal distribution Week 6: normal distribution Week 12: normal distribution	Unpaired *t* test	Week 1: –4.60 to 5.50 Week 6: –4.96 to 0.97 Week 12: –7.66 to 0.69
h. PPr	Week 1: normal distribution Week 6: normal distribution Week 12: normal distribution	Unpaired *t* test	Week 1:–0.13 to 0.44 Week 6: –0.12 to 0.66 Week 12: –0.10 to 0.21
i. Mean A_5_/A_1_	Week 1: normal distribution Week 6: normal distribution Week 12: normal distribution	Unpaired *t* test	Week 1: –0.24 to 0.27 Week 6: –0.17 to 0.64 Week 12:–0.09 to 0.29
j. Input resistance	Week 1: normal distribution Week 6: normal distribution Week 12: normal distribution	Unpaired *t* test	Week 1: –2.00 to 1.86 Week 6: –1.12 to 0.75 Week 12: –2.13 to 0.99
k. Resting membrane potential	Week 1: normal distribution Week 6: normal distribution Week 12: normal distribution	Unpaired *t* test	Week 1: –18.72 to 8.75 Week 6: –11.82 to 9.65 Week 12:–11.37 to 7.67
l. Heart rate	Normal distribution	Two-way ANOVA	Baseline: –73.56 to 100.80 24 h: –188.60 to –14.23 Week 6: –94.12 to 80.28 Week 12: –86.80 to 99.64
m. SDNN	Normal distribution	Two-way ANOVA	Baseline: –0.96 to 0.62 24 h: 0.03 to 1.62 Week 6: –0.78 to 0.79 Week 12:
*n*. RMSSD	Normal distribution	Two-way ANOVA	Baseline: –2.63 to 2.11 24 h: –1.87 to 2.97 Week 6: –2.482 to 2.261 Week 12: –0.92 to 0.77

## Results

### Pilocarpine-induced SE as a model of SUDEP

**Figure 1.**
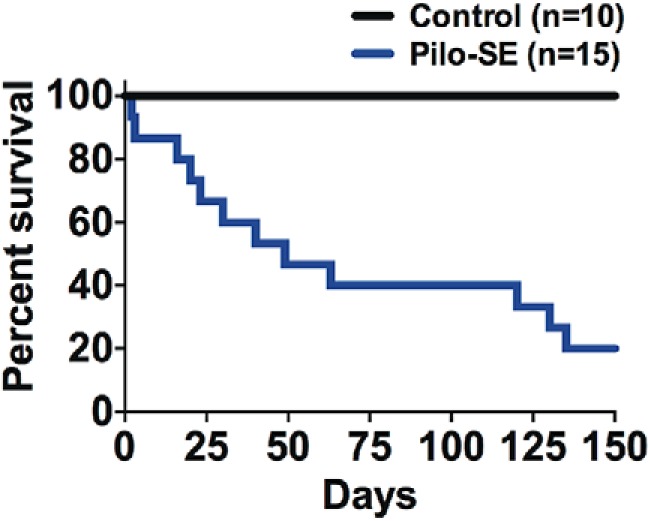
Pilocarpine-induced SE (pilo-SE) increases the risk of sudden death. Pilocarpine-treated mice (Pilo-SE; *n* = 15) have a decreased survival rate (23%) compared to control mice (*n* = 10, 100%; Log-rank Mantel–Cox; *p =* 0.0002). Mice that survived SE died suddenly and unexpectedly at post-SE time points associated with the development of spontaneous seizures.

Spontaneous seizure activity was monitored in a cohort of mice (*n* = 6 control mice; *n* = 6 pilo-SE mice) for 1 wk between 11 and 12 wks post-SE. Similar to previous reports (Shibley and Smith, 2002; Winokur et al., 2004; Hunt et al., 2013), spontaneous seizures were observed during this period in 83% (5 of 6) of pilocarpine-treated mice that survived SE. A separate cohort of mice was monitored for long-term survival after pilocarpine-induced SE. Similar to previous reports (Shibley and Smith, 2002; Winokur et al., 2004), all vehicle-injected mice survived the duration of the monitoring period of 150 days (100%; *n* = 10). Between 1 and 7 d after pilocarpine-induced SE, there was a 13.33% mortality rate (2/15 mice). These mice were not considered to have died of SUDEP, as epilepsy (i.e., with spontaneous seizures) likely had not developed by this time. Of the 13 pilocarpine-treated mice that survived for >7 d post-SE, only three mice (23%) survived to 150 d posttreatment, with no obvious trauma or other incident. All of these mice died 3 wks or more after SE induction; 60% died after >40 d post-SE (Fig. 1). Thus, the survival rate of mice that survived pilo-SE was significantly decreased at 150 d compared with vehicle-treated control mice (log-rank Mantel–Cox; *p =* 0.0002).

### Increased action potential firing in GABAergic NTS neurons from pilo-SE mice is glutamate receptor dependent

**Figure 2.**
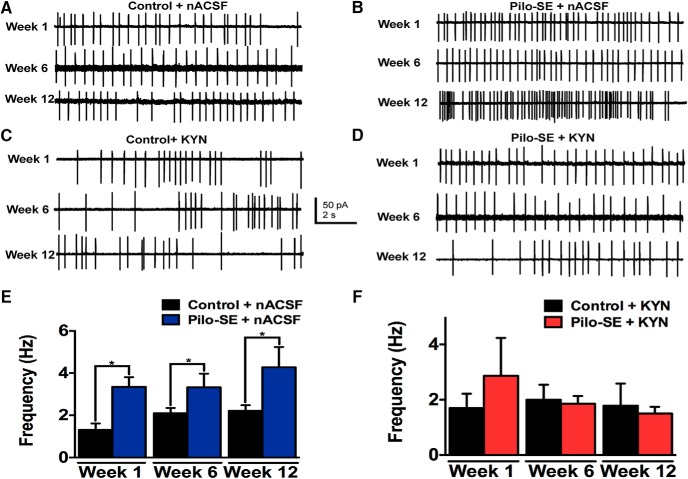
Increased action potential frequency in GABAergic NTS neurons from pilo-SE mice is dependent on glutamate receptor activation. ***A***, Representative traces showing action potential firing (Na^+^ currents) in GABAergic NTS neurons in slices from control mice recorded in normal ACSF (nACSF) at three different time points (i.e., 1, 6, and 12 wks) after vehicle treatment. ***B***, Representative traces showing action potential firing in GABAergic NTS neurons from mice that survived pilo-SE under normal recording conditions (nACSF) at three different time points after SE. ***C***, Representative traces of action potential firing in control mice in the presence of kynurenic acid (KYN; 1 mM) at the same time points. ***D***, Representative traces of action potential firing in the presence of KYN in pilo-SE mice. ***E***, Action potential firing frequency is significantly greater in pilo-SE mice compared with age-matched control mice at 1, 6, and 12 wks posttreatment (unpaired *t* test; *, *p* < 0.05). ***F***, In the presence of KYN, action potential firing frequency in NTS GABA neurons from pilo-SE mice was not significantly different from that of control mice (unpaired *t* test; *p* > 0.05).

Seizure-induced spreading depolarization in the NTS of mice with genetic epilepsies originates in the lateral NTS (Aiba and Noebels, 2015), an area densely comprised of GABAergic neurons (Blessing, 1990; Fong et al., 2005; Glatzer et al., 2007). We hypothesized that GABAergic NTS neurons, most of which receive primary viscerosensory input from the vagus nerve (Glatzer et al., 2007; Bailey et al., 2008), are altered functionally post-SE. On-cell recordings of GABAergic NTS neurons were performed to determine whether spontaneous action potential firing differed between control and pilo-SE mice (Fig. 2*A*,*B*,*E*). One week posttreatment, GABAergic NTS neurons from seven pilo-SE mice displayed significantly higher action potential firing frequency (3.35 ± 0.46 Hz; *n* = 20 cells) compared with NTS GABAergic neurons from seven age-matched control mice (1.32 ± 0.30 Hz; *n* = 15 cells; *p* = 0.002). Spontaneous action potential firing in GABAergic NTS neurons was also significantly increased 6 wks after treatment in five pilo-SE mice (3.32 ± 0.65 Hz; *n* = 16 cells) compared with eight age-matched control mice (2.10 ± 0.25 Hz: *n* = 26 cells; *p* = 0.046). Similarly, action potential firing frequency remained significantly greater in GABAergic NTS neurons from seven age-matched pilo-SE (4.27 ± 0.96 Hz; *n* = 24 cells) than seven age-matched control mice (2.21 ± 0.27 Hz; *n* = 23 cells; *p* = .0048) 12 wks posttreatment (Fig. 2*E*). Therefore, action potential frequency was consistently higher in GABAergic NTS neurons in mice that survived SE than in control mice.

To determine whether the increase in firing frequency was due to increased activation of ionotropic glutamate receptors, spontaneous action potential firing was recorded in the presence of the ionotropic glutamate receptor antagonist, kynurenic acid (KYN; 1 mm; Fig. 2*C*,*D*,*F*). In the presence of KYN, the action potential firing frequency in GABAergic NTS neurons from pilo-SE mice was similar to that of control mice 1, 6, and 12 wks posttreatment (week 1, *p =* 0.47; week 6, *p* = 0.83; week 12, *p* = 0.78; Fig. 2*F*). Therefore, the increased action potential firing in GABAergic NTS neurons from mice that survived SE depended on activation of ionotropic glutamate receptors, implicating increased glutamate-mediated, excitatory synaptic drive to these neurons during epileptogenesis.

### GABAergic NTS neurons display increased synaptic excitatory regulation

**Figure 3.**
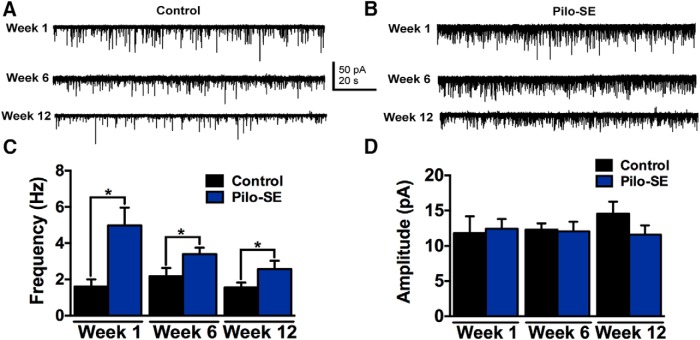
Significantly increased sEPSC frequency in GABAergic NTS neurons from pilo-SE mice. ***A***, Representative traces showing sEPSCs in a GABAergic NTS neuron from control mice 1, 6, and 12 wks posttreatment. ***B***, Representative traces showing sEPSCs in a GABAergic NTS neuron from pilo-SE mice 1, 6, and 12 wks posttreatment. ***C***, sEPSC frequency is significantly higher in GABAergic NTS neurons from pilo-SE mice compared with control mice 1, 6, and 12 wks posttreatment (unpaired *t* test; *, significant). ***D***, sEPSC amplitude is not significantly different (unpaired *t* test; *p* > 0.05) between control and pilo-SE mice at any time point.

An increase in spontaneous action potential firing in GABAergic NTS neurons from pilo-SE mice could occur because of altered intrinsic and/or synaptic properties. To determine whether intrinsic properties were altered post-SE, we measured the resting membrane potential and input resistance in GABAergic NTS neurons and found that there were no significant differences between control and pilo-SE mice at any time point (Table 2). Because firing rate differences were abrogated by KYN, we hypothesized that excitatory glutamatergic synaptic input was increased after pilo-SE. To test this hypothesis, whole-cell patch-clamp recordings were used to examine the frequency and amplitude of spontaneous and miniature EPSCs (sEPSCs and mEPSCs) in GABAergic NTS neurons from age-matched control and pilo-SE mice (Fig. 3). One week posttreatment, sEPSC frequency in GABAergic NTS neurons from six pilo-SE mice (4.98 ± 0.98 Hz; *n* = 12 cells) was significantly greater than seven control mice (1.61 ± 0.40 Hz; *n* = 10 cells; *p* = 0.007). The increased sEPSC frequency was also seen at 6 wks post-SE (five control mice: 2.17 ± 0.46 Hz, *n* = 9 cells; seven pilo-SE mice: 3.40 ± 0.36 Hz, *n* = 13 cells, *p* = 0.045) and 12 wks posttreatment in seven pilo-SE mice (2.57 ± 0.46 Hz; *n* = 15 cells) compared with 11 age-matched control mice (1.55 ± 0.27 Hz; *n* = 20 cells; *p* = 0.03; Fig. 3*C*). There was no significant difference in sEPSC amplitude at any time point posttreatment (week 1, *p* = 0.82; week 6, *p* = 0.89; week 12, *p* 0.2; Fig. 3*D*). Therefore, glutamate release onto NTS GABAergic neurons was increased after pilo-SE, and this increased release persisted for at least 3 months post-SE.

**Table 2.** Resting membrane potential and input resistance of GABAergic NTS neurons in mice that survived SE is not significantly different from age-matched control mice at any time point (unpaired *t* test; *p* > 0.05)

Time	Input resistance (MΩ)		Resting membrane potential (mV)	
	Control	Pilo-SE	Control	Pilo-SE
Week 1	1160 ± 600	1590 ± 490	–47.65 ± 5.35	–51.69 ± 4.25
Week 6	1369 ± 346	1180 ± 293	–52.23 ± 3.69	–53.32 ± 3.63
Week 12	2477 ± 611	1905 ± 429	–55.93 ± 3.31	–57.57 ± 3.33

**Figure 4.**
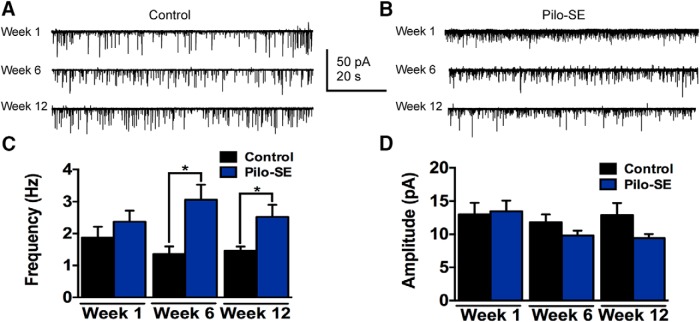
Significantly increased mEPSC frequency in GABAergic NTS neurons from pilo-SE mice. ***A***, Representative traces showing mEPSCs in a GABAergic NTS neuron from a control mouse 1, 6, and 12 wks posttreatment. ***B***, Representative traces showing mEPSCs in a GABAergic NTS neuron from a pilo-SE mouse 1, 6, and 12 wks posttreatment. ***C***, mEPSC frequency is significantly higher in GABAergic NTS neurons from pilo-SE mice compared with control mice at 6 and 12 wks posttreatment (unpaired *t* test; *, *p* < 0.05). ***D***, mEPSC amplitude is not significantly different between control and pilo-SE mice at any time point (unpaired *t* test).

The hypothesis that the increased release of glutamate depended on action potentials in afferent neurons within the slice was tested by measuring the frequency and amplitude of mEPSCs in the presence of tetrodotoxin (TTX; 1 μm), which was added to aCSF to block Na^+^ channels and prevent action potential firing (Fig. 4). Unlike for sEPSCs at 1 wk posttreatment, mEPSC frequency was not significantly increased in five pilo-SE mice (2.37 ± 0.35 Hz; *n* = 9 cells) compared with three control mice (1.88 ± 0.34 Hz; *n* = 8 cells; *p* = 0.33). Miniature EPSC frequency was significantly increased, however, in GABAergic NTS neurons at 6 wks (five control mice: 1.36 ± 0.24 Hz, *n* = 15 cells; six pilo-SE mice: 3.10 ± 0.47 Hz, *n* = 13 cells, *p* = 0.003) and 12 wks (five control mice: 1.46 ± 0.13 Hz, *n* = 15 cells; seven pilo-SE mice: 2.52 ± 0.38 Hz, *n* = 12 cells; *p* = 0.007; Fig. 4*C*). Miniature EPSC amplitude in GABAergic NTS neurons from control and pilo-SE mice was not significantly different at any time point posttreatment (week 1, *p* = 0.85; week 6, *p* = 0.18; week 12, *p* = 0.10; Fig. 4*D*). Therefore, glutamate release was increased in GABAergic NTS neurons after pilo-SE, and the increase detected after 6 wks did not depend on action potential firing in glutamatergic neurons contained within the slice preparation, suggesting that changes at the level of the synaptic terminals contributed to the development of altered glutamate release in the NTS during epileptogenesis.

### Primary vagal afferent input to GABAergic NTS neurons was not altered in pilo-SE mice

**Figure 5.**
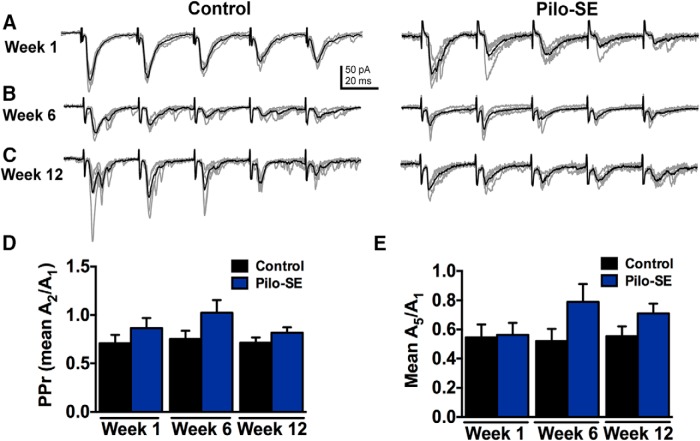
PPr and frequency-dependent depression are unaltered in pilo-SE mice. ***A***, Representative traces of eEPSC responses in GABAergic NTS neurons 1 wk posttreatment from control and pilo-SE mice. ***B***, Representative traces of eEPSC responses in GABAergic NTS neurons 6 wks posttreatment from control and pilo-SE mice. ***C***, Representative traces of eEPSC responses in GABAergic NTS neurons 12 wks posttreatment from control and pilo-SE mice. ***D***, The PPr was not significantly different between control and pilo-SE mice at any time point posttreatment (unpaired *t* test; *p* > 0.05). ***E***, The ratio of the 5th response amplitude to that of the 1st response was also not significantly altered in pilo-SE mice at any time point (unpaired *t* test; *p* > 0.05).

The increase in mEPSC frequency in GABAergic NTS neurons from pilo-SE mice suggests an increase in the probability of presynaptic glutamate release, possibly including from vagal afferent terminals. Nerve terminals of viscerosensory primary vagal afferents synapse directly onto second-order sensory NTS neurons, including GABAergic neurons, the majority of which receive primary vagal input (Glatzer et al., 2007; Bailey et al., 2008). Synaptic responses evoked after stimulating vagal afferents exhibit paired-pulse inhibition and frequency-dependent depression (Miles, 1986; Doyle and Andresen, 2001; Glatzer and Smith, 2005; Bailey et al., 2008). We therefore tested the hypothesis that glutamate release from primary vagal afferents was enhanced in pilo-SE mice by measuring synaptic responses to stimulation of the ST in GABAergic NTS neurons. Examples of responses in NTS GABA neurons to repetitive stimulation of the ST in each group are shown in Fig. 5. One week posttreatment, the PPr was not significantly different between three control mice (0.71 ± 0.07; *n* = 8 cells) and three pilo-SE mice (0.87 ± 0.10; *n* = 8 cells; *p* = 0.27). At 6 wks posttreatment, the PPr was not significantly altered in four pilo-SE mice (0.1.02 ± 0.13; *n* = 8 cells) compared with five control mice (0.75 ± 0.14; *n* = 5 cells; *p* = 0.16). There was also no significant difference in the PPr at 12 wks posttreatment between five control mice (0.71 ± 0.06; *n* = 11 cells) and six pilo-SE mice (0.82 ± 0.06; *n* = 14 cells; *p* = 0.12; Fig. 5*D*). Thus, the increased glutamate release in GABAergic NTS neurons that developed after pilo-SE was likely not due to modification of synaptic release probability in vagal afferents.

Although changes in the PPr after SE survival are an indicator of alterations in the releasable vesicle pool (Schild et al., 1995; Zucker and Regehr, 2002; Pamidimukkala and Hay, 2004), frequency-dependent depression provides insight into synaptic communication between the vagal afferent fibers and GABAergic NTS neurons that may rely on additional mechanisms (Chen et al., 1999; Atwood and Karunanithi, 2002; Kline, 2008; Zhao et al., 2015). Frequency-dependent depression is a common characteristic of second-order NTS neurons receiving viscerosensory afferent input and has been hypothesized to contribute to central adaptation during cardiovascular and respiratory reflexes (Miles, 1986; Doyle and Andresen, 2001; Kline et al., 2005; Glatzer et al., 2007; Bailey et al., 2008; Kline, 2008).

We also tested the hypothesis that frequency-dependent depression was altered in mice that survived SE by analyzing the amplitude ratios of the 5th to the 1st eEPSC in a train (Fig. 5*F*). Similar to the PPr analysis, there was no significant difference between three control mice 1 wk (0.55 ± 0.09; *n* = 8 cells) and three pilo-SE mice (0.56 ± 0.08; *n* = 8 cells; *p* = 0.89), 6 wks (control: 0.52 ± 0.08; *n* = 5 cells; pilo-SE: 0.78 ± 0.12; *n* = 8 cells; *p* = 0.14), or 12 wks posttreatment (control: 0.56 ± 0.07; *n* = 11 cells; pilo-SE: 0.71 ± 0.07; *n* = 14 cells; *p* = 0.31; Fig. 5*F*). These data are consistent with the hypothesis that release properties at vagal afferent synapses with GABAergic NTS neurons are not altered after pilo-SE.

### Heart rate and heart rate variability in mice surviving SE is not altered long-term

In chemoconvulsant-induced SE models of acquired TLE in rats, changes in cardiac rhythmicity that may reflect plasticity of either central or peripheral vagal regulatory function or cardiac remodeling are detected coincident with epileptogenesis (Metcalf et al., 2009b; Bealer et al., 2010; Biet et al., 2015). We examined mouse ECG activity for changes in heart rate and heart rate variability over time after to assess whether ongoing cardiac rhythms were altered following SE. Table 3 describes heart rate and two measures of heart rate variability in six control mice and eight pilocarpine-treated mice that survived SE. Heart rate was significantly increased in mice that survived SE compared with their heart rate 24 h before treatment (baseline, 525.8 ± 22.02 bpm; post-SE, 636.1 ± 39.12 bpm; *n* = 8 mice; *p* = 0.018). This was also true for the SDNN, a measure of heart rate variability. The SDNN was significantly increased 24 h post-SE (baseline, 10.77 ± 0.21 ms; posttreatment, 9.85 ± 0.34 ms; *p* = 0.038). No significant differences were detected at any other time points after SE induction in these same mice, nor were any differences detected over the 12-wk recording period in the RMSSD (two-way ANOVA; *F*(3,46)= 0.135; *p* = 0.939; Table 3).

**Table 3.** Heat rate and heart rate variability (HRV) in mice that survived SE.

Time	Control (*n* = 6)			Pilo-SE (*n* = 8)		
	Heart rate (bpm)	SDNN (ms)	RMSSD (ms)	Heart rate (bpm)	SDNN (ms)	RMSSD (ms)
Baseline	539.42 ± 14.23	10.60 ± 0.13	2.96 ± 0.65	525.8 ± 22.02	10.77 ± 0.21	3.22 ± 0.38
24 h	534.66 ± 8.92	10.68 ± 0.08	3.37 ± 0.51	636.10 ± 39.12	9.85 ± 0.34	2.86 ± 0.59
Week 6	549.96 ± 12.93	10.48 ± 0.13	3.21 ± 1.25	556.90 ± 26.26	10.47 ± 0.23	3.32 ± 0.45
Week 12	530.54 ± 9.28	10.68 ± 0.09	3.12 ± 0.66	524.10 ± 18.19	10.75 ± 0.19	3.27 ± 0.62

Heart rate and the standard deviation of the N-to-N interval (SDNN) were increased 24 h after SE, but no differences were detected at other time points (heart rate: two-way ANOVA, *F*(3,46) = 2.52, *p* = 0.069; SDNN: two-way ANOVA, *F*(3,46) = 2.25, *p* = 0.094). The root mean squared of the standard deviation (RMSSD) was not significantly different at any time point (two-way ANOVA, *F*(3,46) = 0.135, *p* = 0.939).

## Discussion

The present study investigated survival rates and changes in GABAergic NTS neuron function in mice that survived pilo-SE. Mice that died within 1 wk of SE were considered to have failed to recover from SE and therefore not to have died of SUDEP, since they likely did not have epilepsy. Of the mice that survived the first week after pilocarpine-induced SE, just 23% survived to 150 d post-SE, whereas 100% of control mice survived for the duration of the study. Patients with longstanding epilepsy characterized by frequent generalized tonic-clonic seizures that are relatively poorly controlled are at highest risk of SUDEP (Surges and Sander, 2012; Tolstykh and Cavazos, 2013; Massey et al., 2014; Thurman et al., 2014; Dlouhy et al., 2016). SUDEP risk in patient populations with relatively rare genetic epilepsies such as Dravet syndrome, which accounts for ∼3% of patients with epilepsy, is high (Wu et al., 2015), and many studies have been aimed at elucidating the causes of SUDEP in models of genetic epilepsies. Patients with TLE represent ∼60% of all epilepsies, however, and seizures are medically intractable in about one-third of TLE patients, making this the largest epilepsy patient population at risk of SUDEP. The pilocarpine-induced SE model in mice represents a consistent and highly replicable TLE model in which mice develop spontaneous seizures within a few weeks after recovery from SE (Shibley and Smith, 2002; Winokur et al., 2004; Scorza et al., 2009; Bhaskaran and Smith, 2010a, b). A large proportion of the mice that survived pilocarpine-induced SE died suddenly and unexpectedly at time points corresponding with the development of spontaneous seizures (Shibley and Smith, 2002; Winokur et al., 2004; Bhaskaran and Smith, 2010b), promoting this mouse as a reasonable model of SUDEP in TLE. Pilocarpine plasma and brain levels peak in the minutes after injection and fall to almost zero by 2 h postinjection; it is therefore doubtful that the single exposure to pilocarpine itself is responsible for our findings, which were measured days to months after injection (Römermann et al., 2015). Additionally, microinjection of muscarinic receptor agonists in the NTS alters function for 4–6 min after application without sustained changes (Sundaram et al., 1988). Thus, decreased survivability and increased NTS circuit excitability likely develop coincident with epileptogenesis in this model, rather than as a result of brief exposure to the muscarinic agonist.

Sudden unexpected death has been documented in mouse models of genetic epilepsy (Goldman et al., 2009; Glasscock et al., 2010; Cheah et al., 2012; Aiba and Noebels, 2015), and unexpected death has been noted anecdotally in models of acquired TLE. In murine K_v_1.1*^-/-^* and Dravet syndrome genetic epilepsy models, mice begin having seizures by approximately the third week of life, and most animals do not survive past postnatal day 90 (Cheah et al., 2012). These models use genetically mediated ion channel derangement to induce epilepsy, and the channelopathies could themselves increase the likelihood of sudden death. They can also result in altered electrical properties of cardiomyocytes, complicating interpretations of the contribution of the effects of seizures—versus the channelopathy itself—to death (Auerbach et al., 2013). Conversely, seizures in the pilocarpine-induced SE model induce reactive neuroplasticity, including ion channel and synaptic reorganization in cortical structures (Shibley and Smith, 2002; Su et al., 2008; Metcalf et al., 2009b; Bealer et al., 2010; Guo et al., 2013), and the present results indicate they also induce remodeling in brainstem neurons or circuits, which could contribute to central autonomic dysregulation. After the initial post-SE period, heart rate and HRV were not affected in this mouse model, but cardiac arrhythmias have been detected in rats with acquired epilepsy (Powell et al., 2014). An increase in baseline HR that coincided with sympathovagal imbalance has been described in rats 2 wks after pilocarpine injection, before the development of spontaneous seizures (Metcalf et al., 2009a; Bealer et al., 2010). Although similar changes were not detected in the mouse model of TLE used here, further work is necessary to determine whether seizure-related peripheral changes in cardiorespiratory function accompany epileptogenesis in mice, perhaps using isolated hearts to limit the influence of central autonomic regulatory mechanisms (Powell et al., 2014). Our results are consistent with the hypothesis that central autonomic plasticity develops during epileptogenesis in mice, regardless of any potential for cardiac remodeling. Given the critical importance of the vagal complex in regulating cardiac and respiratory reflex function, the development of increased excitability in the NTS during epileptogenesis could reasonably be predicted to increase the propensity for SUDEP in pilocarpine-treated mice.

The vagal complex in the caudal brainstem controls autonomic output to thoracic and most abdominal viscera. Within the vagal complex, GABAergic neurons in the NTS receive, filter, and integrate viscerosensory information regarding cardiorespiratory function and modulate both vagal and sympathetic tone. Neuroplasticity in the vagal complex occurs in a variety of diseases (Mei et al., 2003; Zsombok and Smith, 2009; Bach et al., 2015) and these neurons also displayed functional changes weeks to months after SE. GABAergic NTS neurons displayed significantly and chronically increased spontaneous action potential firing after SE. Significant differences in the passive membrane properties of GABAergic NTS neurons in pilo-SE mice were not detected, but the increase in excitability was accompanied by increased glutamate release, evidenced by significantly higher sEPSC and mEPSC frequency versus age-matched controls. Notably, age-related increases in NTS neuron excitability have been documented (Johnson and Felder, 1993), so all comparisons made here were between age-matched groups. The increased activity was eliminated when ionotropic glutamate receptors were blocked, providing further evidence that long-term changes in synaptic function are associated with epileptogenesis in this model.

The increased glutamate release shortly after pilo-SE was action potential dependent, suggesting an initial increase in excitability of local interneurons. Action potential–independent release, however, was significantly increased by 6 wks, suggesting the development of altered presynaptic release properties or formation of new synapses in the NTS during epileptogenesis. Most GABAergic NTS neurons receive direct vagal input (Glatzer et al., 2007; Bailey et al., 2008), evidenced by eEPSCs with constant response latency (i.e., synaptic jitter <0.2 ms) after TS stimulation. Reduced expression of K^+^ channels in the vagus nerve of K_v_1.1*^-/-^* mice with epilepsy has been reported (Goldman et al., 2009; Glasscock et al., 2010, 2012), and seizure-induced K^+^ channel remodeling in vagal or other afferents could contribute to the increase in glutamate release onto the GABAergic NTS neurons in pilocarpine-treated mice. However, changes in synaptic release properties of vagal afferent terminals were not detected. In addition to synaptic vagal afferent input, these neurons receive glutamatergic synapses originating from local NTS neurons and from other brain areas (Nishimura and Oomura, 1987; Zhang et al., 1999; Glatzer et al., 2007), consistent with the hypothesis that synaptic reorganization of central neurons contributes to increased glutamate release in the NTS during epileptogenesis.

The cellular mechanisms underlying the increased glutamate release and enhanced excitability of NTS GABA neurons have yet to be elucidated, but the increase in synaptic excitation is reminiscent of the synaptic rearrangement that occurs in cortical inhibitory interneurons during epileptogenesis (Hunt et al., 2011; Zhang et al., 2011) and is consistent with dysregulation of autonomic control of the thoracic and abdominal viscera. Increased synaptic excitation of GABAergic NTS neurons would be expected to inhibit parasympathetic motor output and suppress autonomic reflex responses in pilo-SE mice. Because NTS neurons also project to neurons that inhibit medullary sympathetic circuits (Card et al., 2006), increased activity might also chronically disinhibit sympathetic motor output. Respiratory centers receiving input from NTS neurons with altered excitability may also be affected (Stornetta and Guyenet, 1999).

The chronic increase in glutamate-mediated cellular excitability after SE may also make GABAergic NTS neurons more susceptible to sodium channel inactivation in the event of excessive depolarization, as can occur if seizures spread to this brainstem area (Aiba and Noebels, 2015). Spreading depression and depolarization block have been well studied in cortical neurons and implicated in the pathophysiology of migraine and stroke (Dreier and Reiffurth, 2015; Dreier et al., 2015). NTS neurons are normally resistant to spreading depression (Somjen, 2001; Dreier and Reiffurth, 2015), but focal cortical seizures in mice with epilepsy induced spreading depolarization in the NTS under conditions of metabolic deprivation, which was followed by cardiorespiratory collapse and sudden death in genetic epilepsy models (Aiba and Noebels, 2015). Spreading depression is typically initiated in the lateral NTS (Aiba and Noebels, 2015), an area of the nucleus that receives inspiratory vagal afferent input from the lung (Donnelly et al., 1989) and is enriched in GABAergic neurons (Blessing, 1990; Fong et al., 2005; Glatzer et al., 2007). The elevated synaptic excitability in GABAergic NTS neurons in mice that survived pilocarpine-induced SE is consistent with an increased propensity for depolarization block and action potential inactivation in these neurons, which could increase the likelihood that depolarization block and spreading depression could evolve in the NTS (Haller et al., 2001; Larrosa et al., 2006; Sawant-Pokam et al., 2017). Whereas cortical seizures that spread to the NTS can evoke spreading depolarization associated with SUDEP, other coordinated input to the nucleus, such as that which occurs during vagal reflex initiation, might also render the region susceptible to spreading depolarization in mice with TLE. GABAergic NTS neurons play a critical role as mediators of cardiac, respiratory, and baroreceptor reflexes (Andresen and Kunze 1994; Kanter et al., 1995; Wang et al., 2001a, b; Zoccal et al., 2014). Because we did not see any cardiac-specific phenotypes in the mice with pilocarpine-induced TLE, we hypothesize that the increase in NTS neuron excitability leads to an increased propensity for depolarization block and spreading depression centrally, resulting in sudden death under specific conditions (Aiba and Noebels 2015). Notably, this is not necessarily superimposed on chronic changes in cardiac function in this model. These central mechanisms may lead to aberrant baroreceptor or cardiorespiratory reflexes in the pilo-SE mice, but intrinsic changes in cardiac function may not be expressed under nominal conditions.

The present results show that mice that survive SE are susceptible to SUDEP after several weeks. Our findings are consistent with the hypothesis that glutamate release is persistently elevated in the NTS after SE, evidenced by an increase in glutamatergic synaptic input to GABAergic NTS neurons and a corresponding increase in neuronal activity. Chronically increased activity in GABAergic NTS neurons would be expected to impact parasympathetic or sympathetic tone, autonomic reflexes, including cardiorespiratory reflexes, and may underlie seizure-induced depolarization block and spreading depression in the nucleus, leading to cardiorespiratory collapse and SUDEP. Our results also suggest multiple components contributing to the altered excitation of NTS GABA neurons, including an initial increase in glutamate release driven by action potentials in local neurons and a delayed, persistent increase in presynaptic glutamate release from synaptic terminals of central neurons. These changes likely involve seizure-induced synaptic or channel reorganization within the central vagal system. Although the mechanistic cause of SUDEP per se has been debated (Surges and Sander, 2012; Ryvlin et al., 2013; Aiba and Noebels, 2015), it is most likely not due to a single etiology such as cardiac changes, at least in mice with TLE. We posit that in TLE, SUDEP may result from multiple factors (e.g., cardiac or respiratory failure), and the triggers for these are superimposed on dysregulated NTS circuits. Understanding the cellular changes in the NTS that are associated with seizures may prompt the development of predictive biomarkers for SUDEP in those populations most at risk, and eventually therapies to prevent SUDEP.

## Synthesis

Reviewing Editor: Quentin Pittman, University of Calgary Decisions are customarily a result of the Reviewing Editor and the peer reviewers coming together and discussing their recommendations until a consensus is reached. When revisions are invited, a fact-based synthesis statement explaining their decision and outlining what is needed to prepare a revision will be listed below. The following reviewer(s) agreed to reveal their identity: Heidi Grabenstatter

Both reviewers and I agree that this is an interesting manuscript addressing a challenging subject. The reviewers, (who had reviewed your original submission) thought you did a good job of responding to their comments. They have made a few suggestions for further revision, the most significant of which is to provide a bit more detail as outlined by reviewer 1.

I will deal with your revised manuscript myself without further external review and look forward to seeing it published as soon as you can submit the revised manuscript.

Reviewer 1:

Additions have been made detailing the methods used to collect and analyze ECG data. Please share with readers how the ECG data was acquired (beyond the name of the hardware and software). The heart rate variability analysis is conducted in the time domain (i.e., SDNN and RMSSD measures) and not in the frequency domain (i.e., LF/HF ratio is often used to assess parasympathetic and sympathetic changes). For these measures, different frequency ranges are used for mice vs. rats. For the heart rate variability measures that were conducted, what sampling rate was used and how was the data filtered? An AES/ILAE working group is introducing Common Data Elements related to measurement of various physiological parameters of adult rodents to include ECG (in press). Considering your group is one of the first to acquire this type of data from a mouse model of TLE, a full understanding of your methods is important to this work and future work in this area.

Reviewer 2:

The authors are trying to shed some light on the question of how epilepsy causes cardio-respiratory dysregulation that contributes to SUDEP. This is an inherently challenging question to answer. The authors do a reasonable job in designing these experiments to address the question. While there is still a sense that there is some lost opportunity to better correlate some of the changes they see in neuronal excitability with seizures and cardiac changes, it seems that this could be addressed in future studies. Again, the questions addressed in this manuscript are well-reasoned and timely, the experiments are well-designed and seemingly well-executed, and the manuscript is clearly organized and written. The authors were responsive to the previous reviews and have revised the manuscript such that the discussion and conclusions are now well supported by their data.

Minor concerns:

1. Line 92, “with epilepsy” should be added after “individual”.

2.Line 180-181, how was the “six hours during the week” distributed? E.g. one six-hours evaluation period, six one-hour evaluation periods, etc.

3. Line 316 and 319, change “remained” to “was” or “was also”. Saying “remained” implies that the same slices were studied again.

4. Line 348, change “maintained” to “also seen” or something equivalent. Same rationale as in #3.

5. Line 390, add “Six weeks post-treatment,” before “[t]he PPr...”.

6. Line 455, change “Smith, 2010b, a” to “Smith, 2010a, b”.

7. Line 488, change “mode” to “model”.

## Author Response

Reviewer 1:

Additions have been made detailing the methods used to collect and analyze ECG data. Please share with readers how the ECG data was acquired (beyond the name of the hardware and software). The heart rate variability analysis is conducted in the time domain (i.e., SDNN and RMSSD measures) and not in the frequency domain (i.e., LF/HF ratio is often used to assess parasympathetic and sympathetic changes). For these measures, different frequency ranges are used for mice vs. rats. For the heart rate variability measures that were conducted, what sampling rate was used and how was the data filtered? An AES/ILAE working group is introducing Common Data Elements related to measurement of various physiological parameters of adult rodents to include ECG (in press). Considering your group is one of the first to acquire this type of data from a mouse model of TLE, a full understanding of your methods is important to this work and future work in this area.

Author Response: Additions have been made to the methods section "ECG Telemetry" to provide more details on how the data were sampled and filtered. Specifically: "Data were acquired at a sampling rate of 1000 Hz, which is the standard rate used for mice and results in smoother physiological signal when the ECG waveforms are graphed; the telemetry device used a factory preset sampling rate of 200 Hz. No low pass or high pass filtering was applied during data acquisition. For ECG waveform analysis, the software was set to use a 40% QRS detection threshold (percent of the largest derivative peak in a QRS segment resulting in an R that satisfies the minimum heart rate criteria), a minimum R deflection of 0.25 mV, a maximum heart rate of 1000 bpm, and a minimum heart rate of 80 bpm."

Additional information regarding mouse care was also added: "During recovery, mice were housed in an AALAC approved satellite facility in which all ECG recordings were conducted, where they remained for the duration of the 12-week recording period. In order to minimize disturbance and stress to the animals, implanted telemeters were switched on 24 hours prior to each data collection period."

Information regarding the specific methods used to calculate RMSDD and SDNN has also been added: "The SDNN was calculated by taking the square root of the averaged NN interval. The RMSSD was calculated with the following steps: 1) The difference between the NN interval and delayed NN interval was squared; 2) the squared difference was summed; 3). the number of NN intervals was counted; and 4) the sum of the difference squared was divided by the count of NN intervals. RMSSD is reported as the square root of this value."

Reviewer 2:

Minor concerns:

Reviewer 2 Comment 1: Line 92, “with epilepsy” should be added after “individual”.

Author Response: Corrected as suggested.

Reviewer 2 Comment 2: Line 180-181, how was the “six hours during the week” distributed? E.g. one six-hours evaluation period, six one-hour evaluation periods, etc.

Author Response: The monitoring was conducted in two hour sessions, 3 non-consecutive days per week. The methods have been modified to include this information.

Reviewer 2 Comment 3: Line 316 and 319, change “remained” to “was” or “was also”. Saying “remained” implies that the same slices were studied again.

Author Response: The word "remained" has been changed to "was also" in both instances.

Reviewer 2 Comment 4: Line 348, change “maintained” to “also seen” or something equivalent. Same rationale as in #3.

Author Response: "maintained" has been changed to "also seen".

Reviewer 2 Comment 5: Line 390, add “Six weeks post-treatment,” before “[t]he PPr...”.

Author Response: Corrected as suggested

Reviewer 2 Comment 6: Line 455, change “Smith, 2010b, a” to “Smith, 2010a, b”.

Author Response: The citation has been revised here and in one other instance.

Reviewer 2 Comment 7: Line 488, change “mode” to “model”.

Author Response: Corrected as suggested.
